# Scaling genomic reanalysis to unlock diagnoses and transform rare disease care

**DOI:** 10.1016/j.xhgg.2026.100582

**Published:** 2026-02-18

**Authors:** Shira Rockowitz, Wanqing Shao, Courtney French, Tina K. Truong, Jacob Hagen, Rylee McGonigle, Alexa Geltzeiler, Beth Sheidley, Lacey Smith, Alissa M. D’Gama, Mira Irons, Janet Chou, Joan Stoler, Amy Kritzer, Lance Rodan, Akiko Shimamura, Olaf Bodamer, Stephanie Sacharow, Janet S. Soul, Siddharth Srivastava, Amy Roberts Kennedy, Aya Abu-El-Haija, Abbe Lai, Heather Olson, Jane Juusola, Erin Ryan, Bethany Friedman, Anupama Singh, Cliff Li, Rittika Mallik, Gwendolyn Strickland, Gillian Prinzing, Alisa Mo, Anne O’Donnell-Luria, Jeff Bolton, Philip M. Boone, William Brucker, Michael Duyzend, Sonal Mahida, David T. Miller, Jacklyn Omorodion, Jeanette Petit, Jonathan Picker, Annapurna Poduri, Colleen Carlston, Monica H. Wojcik, Piotr Sliz, Wendy K. Chung

**Affiliations:** 1 Children’s Rare Disease Collaborative, Boston Children’s Hospital, Boston, MA, USA; 2 Division of Genetics and Genomics, Boston Children’s Hospital, Boston, MA, USA; 3 Department of Pediatrics, Boston Children’s Hospital, Boston, MA, USA; 4 Harvard Medical School, Boston, MA, USA; 5 The Manton Center for Orphan Disease Research, Boston Children’s Hospital, Boston, MA, USA; 6 Boston Children’s Hospital, Department of Neurology, Boston, MA, USA; 7 Boston Children’s Hospital, Division of Newborn Medicine, Boston, MA, USA; 8 Broad Institute of MIT and Harvard, Cambridge, MA, USA; 9 Boston Children’s Hospital, Division of Immunology, Boston, MA, USA; 10 Department of Hematology and Oncology, Boston Children’s Hospital, Boston, MA, USA; 11 Dana Farber Cancer Institute, Boston, MA, USA; 12 Rosamund Stone Zander Translational Neuroscience Center, Boston Children’s Hospital, Boston, MA, USA; 13 Department of Cardiology, Boston Children’s Hospital, Boston, MA, USA; 14 GeneDx, LLC, Gaithersburg, MD, USA; 15 Division of Developmental Medicine, Boston Children’s Hospital, Boston, MA, USA; 16 Division of Molecular Medicine, Boston Children’s Hospital, Boston, MA, USA; 17 Department of Biological Chemistry and Molecular Pharmacology, Harvard Medical School, Boston, MA, USA

**Keywords:** genomic reanalysis, rare disease diagnostics, exome and genome sequencing, ES/GS, proactive genomic reanalysis, PGR, clinical genomics infrastructure, machine learning in variant prioritization, variant reclassification, scalable genomic medicine, diagnostic yeild improvement, precision medicine implementation

## Abstract

Genomic reanalysis can identify causative variants for rare diseases as patient phenotypes evolve and gene-disease knowledge expands. Despite its diagnostic value, routine reanalysis is limited by clinician capacity, lack of patient follow-up, data silos, cost, and lack of availability of clinical data to testing laboratories that are not obligated to conduct reanalysis. The Children’s Rare Disease Collaborative at Boston Children’s Hospital (BCH) has integrated genomic and phenotypic data from over 15,500 patients into a clinician-facing platform. Leveraging this infrastructure, we developed a Proactive Genomic Reanalysis (PGR) workflow at BCH for clinical sequencing data that is centralized, semi-automated, and clinically integrated. Here, we report initial results and outline required resources and transferable insights applicable to other healthcare settings. Initial pilot implementation, applied to a subset of clinical sequencing patients’ data, revealed practical challenges, notably clinician turnover and patient recontact difficulties. Of 42 patients’ candidate variants discovered by the PGR bioinformatics pipeline and returned to treating clinicians, 33 were determined to have a high suspicion of disease causality and an additional 3 were determined to be candidate variant of uncertain significance. A process to generate reports and return results to patients was initiated when applicable. Although the initial pilot implementation was limited, the PGR bioinformatics pipeline is designed to be utilized iteratively, making reanalysis a continuing process. This work highlights the feasibility and impact of centralized PGR processes and the potential for healthcare institutions to scale genomic reanalysis.

## Teaser

Centralized, semi-automated reanalysis at Boston Children’s Hospital enables ongoing rare disease diagnoses. This proactive, clinician-driven model transforms reanalysis from an occasional event into a dynamic, iterative process integrated with clinical care.

## Introduction

Genomic medicine is enhancing patient care, particularly since methodologies such as exome sequencing (ES) and genome sequencing (GS) have become a part of standard clinical practice and cost-effective diagnostic tools.^1^^,^^2^^,^^3^^,^^4^^,^^5^ Precision medicine interventions depend on accurate genetic diagnoses,^6^ but a substantial portion (55%–67%) of patients with a presumed genetic condition remain without a definitive diagnosis despite ES/GS.^7^ This diagnostic gap is often due to incomplete understanding of genomic variation responsible for the disease, challenges regarding the detection of certain variant types due to the nature of short read ES/GS modalities as well as bioinformatics limitations, limitations in phenotyping younger patients whose disease manifestation is still evolving at the time of testing, and the static nature of traditional laboratory workflows.^8^

Reanalysis of genomic data has emerged as a powerful strategy to address this gap, diagnosing between 2% and 31% of previously non-diagnostic cases after 1–5 years.^9^^,^^10^^,^^11^^,^^12^^,^^13^^,^^14^^,^^15^^,^^16^^,^^17^^,^^18^^,^^19^^,^^20^^,^^21^^,^^22^^,^^23^ Clinician involvement in reanalysis has a large (11.9%) diagnostic impact, improving the interpretation of the clinical context for genes considered to be associated with the patient phenotype.^24^ Although it is well known that reanalysis increases diagnoses, current approaches to clinical testing remain largely static based upon the payment model where reanalysis is not uniformly covered by health insurance but may be offered free of cost according to individual laboratory policies.^25^^,^^26^ Genomic data are analyzed initially at the diagnostic laboratory; subsequently the locus of control is with the clinician to initiate lab reanalysis since they can best assess the patient’s clinical state and relevance of receiving a diagnosis.^27^ Raw genomic data, which could be reanalyzed by clinicians as information is amassed, typically stays with reference laboratories, which generally do not initiate reanalysis unless prompted by a request from the treating clinician. Even reclassification of variants that were already included in the clinical report is not always performed due to implementation complexities.^28^ Proactive reanalysis, defined here as institution-initiated and systematically applied reanalysis, as opposed to clinician-initiated reanalysis, has been shown to have a large additional impact on initially unsolved ES.^21^ Although clinicians perceive automated or semi-automated reanalysis, referring to computational approaches that reduce manual effort, as imparting many clinical and workflow benefits^29^^,^^30^^,^^31^ several challenges impede adoption including: data are separated, with changing clinical information residing at hospitals with clinicians while genomic data generally reside at reference laboratories; there is little documented about potential insurance reimbursement for recurrent reanalysis^25^; and there are no turnkey commercial or research tools available to make the process of reanalysis simple, automatic and scalable.

To date, systematic reanalysis efforts have been primarily focused on cohorts of research participants.^9^^,^^11^^,^^15^ Many of these efforts focused on specific diseases and performed a single bulk reanalysis rather than an ongoing iterative process that reaches individual patients with unique phenotypes. Various approaches to streamline algorithms involved in genomic reanalysis have been reported, but manual processes and large review groups are still core to the workflow.^9^^,^^10^ Although there is a large body of literature regarding the utility of reanalysis in the research space, automatic clinical reanalysis—re-evaluation of existing genomic data within a clinical framework—is rarely practiced. Only one commercial or academic laboratory out of 28 surveyed by Frees et al., practiced any automated reanalysis.^25^ Most (86%) of the laboratories solely relied on clinician-initiated reanalysis and, as a result, clinical reanalysis uptake has been low (<10%).^25^^,^^29^ No reports have described the systematic and standard application of reanalysis to clinical data as part of clinical processes after initial sequencing, nor closed the loop regarding the impact of the evolving clinical findings for patients. While clinician-initiated laboratory reanalysis and research-based reanalysis have contributed to diagnoses at Boston Children’s Hospital (BCH), these approaches remain episodic, resource-intensive, and dependent on individual clinician initiative or research enrollment. In this manuscript, these existing reanalysis pathways are presented to establish the institutional baseline and unmet need that motivated the development of a centralized Proactive Genomic Reanalysis (PGR) workflow.

Here, we describe the development and deployment of a PGR workflow at BCH for clinical sequencing data that is centralized, semi-automated, and clinically integrated. We were able to leverage our established process for the return of raw genomic data to the institution after clinical ES/GS testing and research technologies utilized by the Children’s Rare Disease Collaborative (CRDC) to enable clinicians to use BCH analytic tools to review and interpret prioritized candidate variants for their patients. We describe the development of the PGR bioinformatics pipeline to semi-automatically identify variants associated with patients’ evolving phenotypes at scale, the processes developed to confirm and return those findings to patients, and complexities discovered along the way. We outline necessary resources and transferable insights applicable in other healthcare settings. PGR is designed to empower clinicians, as those most familiar with the patient’s evolving phenotype, to regularly and systematically review possibly relevant variants for all patients. By bridging the gap between laboratory infrastructure and frontline clinical care, this model facilitates a dynamic and responsive approach to genomic medicine. Our approach demonstrates how a centralized, dynamic institution-initiated reanalysis system can be deployed at hospitals that service many patients with genetic conditions to democratize access, enhance clinical utility, and serve as a scalable model for genomic medicine.

## Subjects and methods

### Underlying infrastructure and agreements

The implementation of a collaborative, clinically integrated PGR workflow at BCH was built on previously established pipelines for genomic data return and data processing and analysis. In early 2018, raw sequencing data from Claritas Genomics was returned to the hospital en masse and included 576 probands and 493 relatives. Beginning in 2019, as per the recommendation from the BCH Genomic Consent Taskforce, all raw sequencing data and metadata from the current clinical sequencing provider, GeneDx, has been returned to BCH via establishment of a robust data transfer and integration framework (Figure S1A). This dataset comprises a total of 6,279 probands and 10,825 relatives through May 1, 2025 (47 probands and 75 relatives had sequencing performed at both Claritas Genomics and GeneDx). Leveraging the previously published CRDC genomic analysis infrastructure^32^^,^^33^ enabled rapid deployment of existing methodologies for collecting Human Phenotype Ontology (HPO) terms from the electronic health record (EHR) via natural language processing (NLP) on a monthly basis, harmonizing variant calling data via secondary analysis pipeline DRAGEN, and loading data into established BCH analytic tools for clinicians (Figure S1B). The BCH analytic tools integrate dynamic phenotypic and genomic data and include a suite of genomic analysis tools hosted by GeneDx (distinct from the GeneDx tools used for internal clinical analysis and hereafter called “the GeneDx research platform”), an instance of seqr,^34^ which is hosted at BCH, and REDCap.^34^^,^^35^

### Genomic data analysis training and quality improvement survey

**Figure 1:**
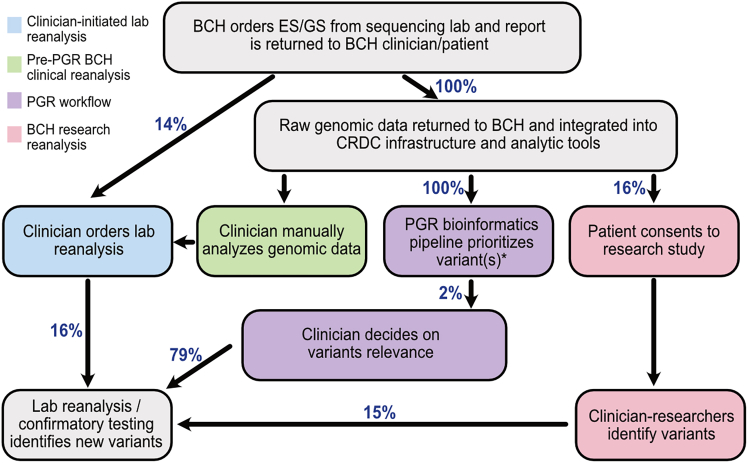
Overview of reanalysis workflows at BCH Clinicians at BCH order ES/GS from the sequencing lab and the initial report is returned to the patient. In 13.6% of patients, a clinical reanalysis from the lab is ordered (blue). A total of 16% of patients with such a reanalysis ordered have findings. The raw data for all patients and relatives included in clinical sequencing are returned from the sequencing laboratories (Claritas Genomics and GeneDx) to BCH and integrated into the CRDC infrastructure and institutional analytic tools. We do not have an estimate of how often clinicians review genomic data (green) as this activity is not routinely tracked but 21% of clinician respondents (7/33) who took the seqr training at BCH (a group highly biased to interest in genomics) said they had previously looked at a patient’s raw genomic data. This activity reflects existing, decentralized clinician expertise and access to analytic infrastructure at BCH; while not a scalable or systematic reanalysis workflow, it provides essential clinical context and human interpretation that inform and complement the centralized PGR process. The PGR bioinformatics pipeline is applied to all data from patients with no reported variants and will soon be expanded to all patients with data returned to BCH (purple), identifying variants in 2% (42/2,144) of patients. A total of 79% (33/42) variants identified were considered by clinicians to be diagnostic. ∗Limited variants were discussed in this article due to the specific nature of the initial pilot implementation of the VS-NN algorithm. We expect that increasing the sensitivity of the PGR bioinformatics pipeline will vastly increase the percentage of patients with identified variants. A total of 16% of patients consent to research studies (e.g., CRDC studies such as the Manton Center^36^) (pink); these research efforts are not evaluated as part of this analysis and are shown solely to illustrate the broader institutional genomic research environment in which PGR operates. French et al.^33^ report that 15% of the patients participating in the CRDC have variants clinically reported.

To facilitate clinician access to their patients’ data for reanalysis (Figure 1, green box), a training session was held to introduce the process. Following this training session, clinicians who attended were granted access to patient genomic data and invited to attend monthly office hours to bring their cases and ask for help. An offer was also extended via email to help with any reanalysis cases. Following these interventions, a survey was sent to those who had signed up for the training session as well as other genetics and neurology department members to assess interest in accessing their patients’ data, comfort level doing so, frequency of ordering genetic testing, previous experience with genomic analysis, and any barriers or concerns with the process (Figure S2).

### Transfer of genomic data and metadata from Claritas

Genomic data (ES hg19 BAM files) were transferred as a one-time data deposit via Amazon Web Services (AWS) to BCH in January of 2018. Metadata were contained in an excel spreadsheet and included Claritas accession to identifier information, test ordered (bone marrow failure panel, neurology panel, ES) for all individuals (*n* = 1,069) and HPO terms for some patients (42/576 probands had HPO terms annotated in the metadata). Variants reported by Claritas were not returned to BCH in a structured format.

### Transfer of genomic data and metadata from GeneDx

Data from 6,279 probands and 10,825 relatives were returned to BCH by GeneDx between April 11, 2019, and May 1, 2025. Genomic data (ES and GS hg19 CRAMs and VCFs) were delivered to BCH via AWS as an ongoing process as sequencing was completed. Metadata returned via REDCap included predicted genetic similarity to reference groups determined by principal-component analysis, mean coverage, sex, kinship scores, GeneDx case identifiers, HPO terms, percent 10× coverage, heterozygosity/homozygosity ratios (including X chromosome-specific metrics), and which family members were sequenced. For each individual, accession numbers, file locations, and file names were recorded. Variant-level data encompassed gene names, predicted effects, HGVS cDNA and protein annotations, clinical interpretations, zygosity, inheritance patterns, and genomic coordinates (hg19), along with variant-specific zygosity across all sequenced family members. A custom extract-transform-load process was developed, deployed, and iteratively refined to translate case-level metadata, where each additional family member or reanalysis review generated a case, into an individual-centric format compatible with the BCH database architecture.

### Analysis of variants reported by GeneDx

Variants were classified by GeneDx in accordance with the American College of Medical Genetics and Genomics (ACMG) guidelines for variant interpretation from Richards et al.^37^: causative pathogenic or likely pathogenic (P/LP), possibly associated P/LP, possibly associated variant of uncertain significance (VUS), and candidate VUS. Causative variants refer to P/LP variants in genes associated with human disease where the disease phenotype aligns with the patient’s reported clinical findings. Possibly associated variants refer to a VUS or P/LP variant in genes that may be associated with the patient’s phenotype but are not clearly diagnostic. Some examples of possibly associated variants include a single P/LP variant in an autosomal recessive (AR) gene where a second variant was not identified, variants classified as VUS in genes that are a moderate fit for the phenotype, and single P/LP variants in a gene that has both AR and autosomal dominant (AD) inheritance but the phenotype is not exactly aligned with the known AD phenotype. Candidate variants refer to a variant in a gene that needs additional evidence to establish whether there is a relationship between the gene and human disease. A total of 234 probands was removed from the analysis because they were not easily mappable to the above categories including instances where the variant was annotated as previously reported (*n* = 183) and 51 probands who were removed due to unclear variant reporting in reanalysis, e.g., a variant appearing in a reanalysis as previously reported that had not been included in the initial analysis. Following these exclusions, 6,045 probands were included in the subsequent analyses. Counts of probands with reported variants are included in Table 1, labeled based on the most impactful variant hierarchically. Probands with no reported variants or variants that do not belong to the above categories, including patients who only had ACMG secondary findings reported, were labeled as “no reported variants.”

**Table 1:** Baseline clinical variant findings, clinician-initiated reanalysis, and PGR pilot results

	Patients with annotated variants in original analysis	Patients reanalyzed by clinical sequencing provider	Patients with changes to their reports after reanalysis by clinical sequencing provider	Patients with variants upgraded through clinician-initiated clinical laboratory reanalysis	Patients with variants downgraded through clinician-initiated clinical laboratory reanalysis	Patients with variants downgraded and upgraded through clinician-initiated clinical laboratory reanalysis	No change to variant after clinician-initiated clinical laboratory reanalysis	Patients reanalyzed by PGR	Patients with variants from PGR	Patients with variants from PGR, high suspicion of disease causality or candidate VUS
Causative P/LP	1,303	34	5	3	2	0	29	N/A	N/A	N/A
Possibly associated P/LP	765	56	10	2	7	1	46	N/A	N/A	N/A
Possibly associated VUS	1,050	92	17	16	1	0	75	N/A	N/A	N/A
Candidate VUS	250	23	6	5	0	1	17	N/A	N/A	N/A
No reported variants (2023+)	1,572	19	5	5	0	0	14	606	4	4
No variant reported (2021–2022)	866	146	20	20	0	0	126	825	12	9
No variant reported (2019–2020)	239	70	13	13	0	0	57	208	4	3
Unknown (Claritas)	529	N/D	N/D	N/D	N/D	N/D	N/D	505	22	20

This table shows the different types of patients with data input into the pilot implementation of the PGR pipeline. N/D, not determined; N/A, not applied.

### EHR metadata

Date of birth, sex, self-reported race and ethnicity, zip code, and type of health insurance were pulled from the EHR for each proband on May 27, 2025. Age at sequencing was calculated as the year of sequencing minus the date of birth. Family structure was calculated as proband only if only the proband had data returned by GeneDx, duo if the proband and one parent had data returned by GeneDx, and trio if the proband and both parents had data returned by GeneDx. Revised combined race and ethnicity category was calculated from self-reported race and ethnicity as defined in Chopra and co-workers.^38^ Zip code was used to categorize by distance from the hospital and was integrated with the Agency for Healthcare Research and Quality’s database on Social Determinants of Health zip code data from 2020^39^ to obtain the median income for the zip code of origin when the zip code was in in the United States. NLP of clinical notes was performed using Clinithink’s CLiX Focus software to extract HPO terms on a recurring monthly basis as described in Rockowitz et al.^32^

### Statistical analysis of EHR data

Two-sided Fisher’s exact tests were run on each categorical variable within the following demographics: age, sex, family structure, revised combined race and ethnicity category, distance to BCH, type of health insurance, median income of the zip code of residence, and variant type (P/LP, VUS, no reported variants) and reanalysis status to identify variables that had significant associations. A total of 102 tests was performed comparing categorical variables to variant type and 34 tests were performed comparing categorical variables to reanalysis status. Benjamini-Hochberg correction was performed on all resulting *p* values to adjust for multiple hypotheses testing.

### Reanalysis annotations in clinical notes

HoundDog,^40^ an internally developed tool at BCH for searching and indexing free text within clinical notes, was used to scan clinical notes to identify patients who had terms related to sequencing (“WES” or “WGS” or “exome” or “genome”) and terms related to reanalysis (“reanalysis” or “re-analysis”). If a patient had a single clinical note with both sets of terms, they were classified as having reanalysis annotated in their clinical notes. A total of 4,975 patients had reanalysis annotated in their clinical notes; 88 patients were excluded because they were not easily mappable to defined categories of GeneDx analysis results (see analysis of variants reported by GeneDx). Of the remaining 4,887 patients, 407 were included in the 440 who underwent clinician-initiated clinical laboratory reanalysis.

### Annotation of reanalyses reported by GeneDx

Probands with clinical reanalysis were identified as those with the original test ordered after April 11, 2019, and having multiple GeneDx case identifiers. The BCH schema only allowed for one proband per family. Cases with changes in family structure or other uncertainty were excluded. Secondary analysis of data from GeneDx’s internal database of reanalysis led to the exclusion of another of 89 probands. For the remaining 440 probands who underwent clinical reanalysis, the labeling system described in analysis of variants reported by GeneDx was applied to the reanalyzed data. Patients were then categorized as having (1) variants that were upgraded or added through reanalysis, (2) variants that were downgraded or removed through reanalysis, (3) variants upgraded/added and variants downgraded/removed, and (4) no change after reanalysis.

### Secondary and tertiary analysis

**Figure 2:**
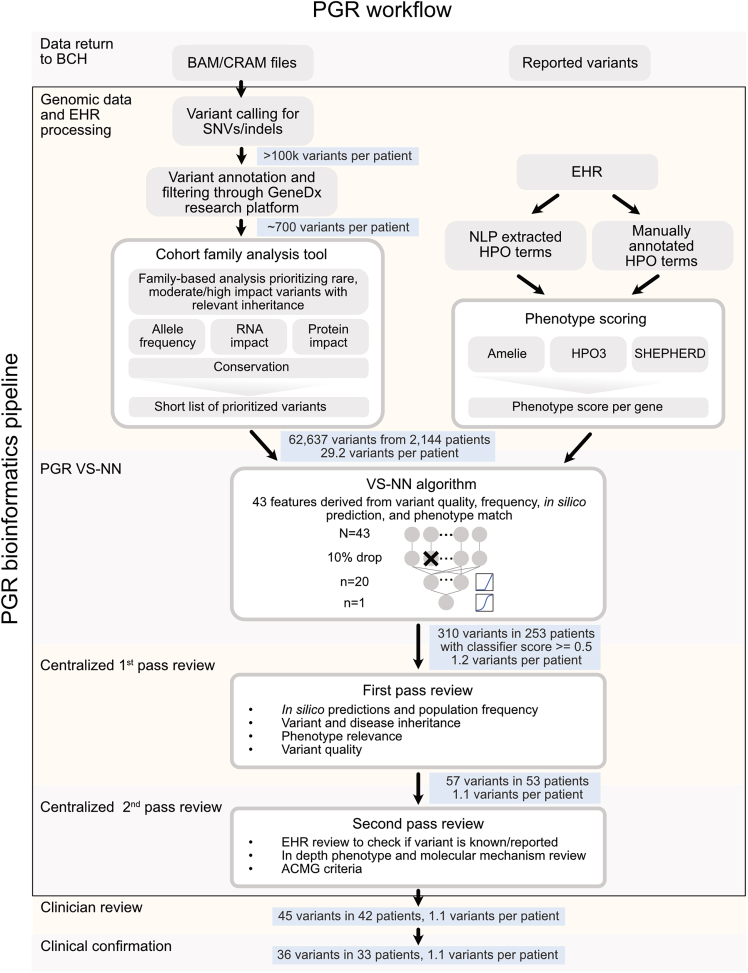
PGR workflow This diagram visualizes the overall PGR workflow, which includes the PGR bioinformatic pipeline (VS-NN algorithm followed by first- and second pass review processes). This figure contains the number of variants remaining after each stage of the workflow.

All ES and GS data were reprocessed using DRAGEN read mapping and variant calling pipelines on human reference genome build hg38 (Figure 2). SNVs and small indels from the standard DRAGEN VCF output were integrated into the GeneDx research platform, which provides mode of inheritance based on joint genotype calling when parental samples are available, variant annotations using Ensemble’s Variant Effect Predictor (VEP),^41^ gnomAD v.2,^42^ dbnsfp,^43^ ClinVar,^34^ OMIM,^44^ HPO,^45^ and a custom variant classification prediction based on the ACMG scoring,^33^ in line with the approaches described in InterVar^46^ and the Bayesian classification framework by Tavtigian et al.^47^ The GeneDx research platform does not provide allele frequency, variant classifications, or phenotype annotations from GeneDx’s proprietary databases, but instead relies on the publicly available sources listed above. Initial filtering restricts variants in the VCF based on variant effect (exonic ±20 base pairs or P/LP in ClinVar^48^), allele frequency across reference databases and internal BCH cohorts, and variant quality (to filter out low-quality variants and regions with low-quality mapping).

### Cohort family analysis

This study used the default cohort family analysis (CFA) filters, which includes the following: HIGH (loss of function) and MODERATE (other protein-changing) VEP impact, allele frequency ≤1.5%, heterozygous count ≤30, homozygous count ≤2, not annotated benign (i.e., not ClinVar benign/likely benign nor GeneDx research platform ACMG auto-classification benign/likely benign or VUS with only benign criteria), and inheritance pattern consistent with *de novo*, compound heterozygous, homozygous, or hemizygous inheritance or otherwise segregating with affected members of the family. For cases where parent data are not available (non-trios), additional filters were used including stronger frequency and impact filters (e.g., high CADD, constraint, or splicing scores) and requiring either patient-gene phenotype overlap or pathogenic annotation of the variant.

### HPO match and phenotype scoring

To speed manual variant review, HPO term matches between the patient’s terms and the terms associated with the gene containing the variant were extracted. Exact term matches along with matches between terms with one ontology level difference were pulled. Additionally, a term information content^49^^,^^50^^,^^51^ cutoff of two and four was employed to highlight more specific and rare term matches. Furthermore, phenotype scoring between the patient’s terms and the terms associated with the gene was performed using HPO3,^49^^,^^50^ AMELIE,^52^ and SHEPHERD.^53^ The resulting term match features and phenotype scores were used as input to a variant-scoring algorithm.

### Variant-scoring algorithm

A total of 2,575 variants (2,154 from clinically ordered sequencing, 421 clinically confirmed findings from research) from 1,844 probands was used to train the variant-scoring neural network (VS-NN) algorithm. Variants that were categorized as pathogenic (*n* = 434), likely pathogenic (*n* = 811), and VUS (*n* = 1,330), were labeled as positive instances. A total of 53,223 variants that passed the CFA filters but were absent from clinical reports for these cases served as negative instances for model training. Each variant was annotated with 43 features, including variant allele fraction, variant depth, variant inheritance, gene inheritance, VEP,^41^ ClinVar clinical significance,^48^ gnomAD v.4 frequency,^42^ CRDC internal frequency, HPO3,^49^^,^^50^ AMELIE,^52^ SHEPHERD,^53^ HPO match,^49^^,^^50^^,^^51^ CADD PHRED,^54^ REVEL,^55^AlphaMissense,^56^ SpliceAI,^57^ MisFit,^58^ S_coef_,^59^ S_het_,^59^ pLI,^60^ and GeneDx research platform ACMG classification.^37^ These features were numerically encoded, standardized, and used as input for the VS-NN algorithm, which outputs a pathogenicity score ranging from 0 to 1. For initial model training, 30% of the dataset was reserved for validation and, after evaluating model performance, the final model was retrained using the full dataset. After training, the VS-NN algorithm was applied to 62,637 variants that passed the CFA filters from 2,144 patients with no reported variants as of July 26, 2024, and variants with a score ≥ 0.5 underwent manual bioinformatician review.

### Pruning HPO analysis

For each patient analyzed, the ancestral hierarchies of the manually collected HPO terms were traced up to the third highest level in the ontological tree. Terms with the same third-level ancestor were grouped into branches representing related phenotypes. The pruning algorithm was run repeatedly, removing one branch each time, to simulate phenotype clusters (e.g., neurological) not yet present earlier in the patient’s history. Following each pruning iteration, the VS-NN algorithm was rerun to assess the effect of branch-level term reduction on downstream variant prioritization and model performance. *p* values comparing different sets of HPO terms in true positives (TPs) and false negatives (FNs) from the validation dataset were calculated using two-sided Wilcoxon rank-sum tests and corrected using the Benjamini-Hochberger method.

### Manual bioinformatician review of the VS-NN algorithm results

Variants from the VS-NN algorithm with a score ≥ 0.5 were screened by a PhD bioinformatician with variant curation training. The bioinformatician verified that *in silico* predictions did not suggest a benign or likely benign classification. Population frequency was evaluated using gnomAD v.4, including a review of all possible variants at the coordinate and summing allele counts (AC) and frequencies (AF) for variants with similar impact. For recessive variants, strict thresholds (gnomAD AF ≤ 0.001 and homozygous/hemizygous count ≤2) were applied, only considering variants with homozygous/hemizygous presence if the phenotype match was strong. For dominant variants not associated with neurodevelopmental disorders, a gnomAD AC ≤ 2 was required unless incomplete penetrance was supported by literature or phenotype match was compelling. For dominant variants linked to neurodevelopmental conditions, variant presence in gnomAD v.3 controls and non-neurodevelopmental populations were examined, removing those with AC > 2 in controls and retaining those with up to 1 in non-neurodevelopmental populations, unless incomplete penetrance was supported by literature. Homozygous/hemizygous presence in gnomAD was not allowed for dominant variants. The variant’s mode of inheritance consistency with the disease inheritance pattern in OMIM was confirmed, if an OMIM record was available. If an OMIM record was not available, AMELIE literature matches and a PubMed search were used to gather information about the mode of inheritance for the disease. The phenotype relevance using OMIM, AMELIE literature matches and the patient’s EHR were then assessed. Variants that only meet OMIM susceptibility to diseases were not returned. Finally, all candidate variants underwent manual quality confirmation in IGV.^61^

### Manual second pass review of VS-NN algorithm results

Variants from the VS-NN algorithm that also passed bioinformatician review were then manually reviewed by a licensed genetic counselor or PhD variant curator with significant ACMG criteria application training for deeper review of genotype-phenotype correlation, gene-disease association, and ACMG variant criteria.^47^^,^^62^ This review was based on available literature, the Clinical Genome Resource (ClinGen^63^) and Gene Curation Coalition (GenCC^64^) for gene-disease validity as well as the Sequencing Variant Interpretation (SVI) frameworks. VUS leaning P/LP per SVI and ACMG criteria and/or had an identified functional assay that could potentially be pursued by the ordering physician and thereby upgrading the pathogenicity were determined to be returnable.

### Establishment of a centralized genomic reanalysis workflow

A centralized genomic reanalysis process was established in 2024, when a strategic working group was convened to develop more scalable approaches to support genetic diagnoses, clinical trials, and clinical implementation of genetic therapies within the Department of Pediatrics and more broadly across BCH. The BCH Genomic Medicine/Genetic Therapies Working Group included clinicians across a wide range of specialties and identified areas of focus where pilot projects could have the biggest impact on patient care. At the time, several reanalysis options were already in place at BCH, including clinician-initiated lab reanalysis (Figure 1, blue box), where the laboratory reanalysis was requested at the clinician’s discretion directly by the clinician who was seeing the patient; clinician manual analysis of genomic data (Figure 1, green box), which was empowered by structured training for clinicians who routinely ordered genetic testing on the BCH genomic analysis tools (Figure S2); and research reanalysis (Figure 1, pink box), which consisted of investigator-initiated Mendelian disease research efforts performed by research teams and included CRDC-affiliated studies such as the Manton Center,^36^ rather than a single standardized research pipeline or institutional workflow. One of the recommendations that emerged from this process was for a pilot project focused on a centralized, automated reanalysis process within the hospital that was clinically meaningful and scalable across varied care settings, leading to the development of the PGR workflow. The initial pilot implementation phase included the development of the semi-automated BCH PGR bioinformatics pipeline to identify potentially diagnostic variants, application of the bioinformatics pipeline on currently available clinical genomic data, and definition of the clinical processes to return the identified variants to clinicians and patients. The initial pilot implementation was conducted between July 2024 and May 2025 and was a multidisciplinary effort among clinicians in multiple departments/divisions, institutional leadership, and the technical team from the CRDC. The centralized working group consisted of clinical and bioinformatics leadership, including PhD bioinformaticians, MD and MD/PhD clinicians with expertise in clinical and molecular genetics and licensed genetic counselors, who together led the development of the PGR bioinformatics pipeline, established variant review and result dissemination workflows and supported clinician engagement. The procedures followed were part of a clinical quality improvement program.

### PGR workflow implementation and pilot context

**Figure 3:**
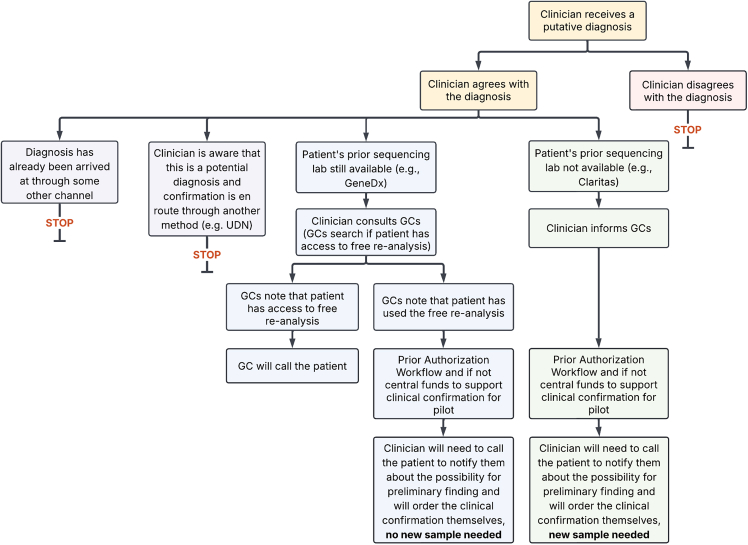
Clinical workflow diagram This flowchart depicts the process followed by the clinician after receipt of a variant of interest from the centralized working group. Genetic counselor indicated by GC in the workflow diagram.

During the pilot phase, the PGR workflow was clinically integrated and operationalized as part of routine clinical processes. Internal reports generated by the centralized working group were reviewed by clinicians and workflows were defined for clinicians to coordinate confirmation of the identified variants. An internal report, which contained one to two variants, was communicated to the clinician who was most familiar with the patient, frequently this was the ordering provider for the initial ES/GS. If the ordering clinician and/or the clinician most familiar with the patient had left BCH, a clinician was identified by their Division/Department. To address provider turnover, the Division of Genetics and Genomics and Department of Neurology, who respectively ordered 64% and 33% of the ES/GS at BCH, established workflows to reassign responsibility to a provider. The expectation was that the clinician would review the internal report and the patient’s medical record holistically to make a determination about whether the variant was a good phenotypic match and cause for the patient’s phenotype (Figure 3). The report (Figure S6) included information to inform the clinician about the variant, such as published allele frequencies in gnomAD, PubMed IDs for relevant publications, and internal ACMG scoring conducted by the second pass reviewer. If the clinician determined that the variant fit the patient’s phenotype and a diagnosis had not already been delivered to the patient, then the clinician would reach out to the patient to coordinate and initiate the variant confirmation by the CLIA lab. The report also contained guidance on how to request confirmation of the variant(s) if one was needed. Patients would be informed of the specific variants only after clinical reanalysis or targeted gene testing was completed. When possible, variants would be confirmed by lab reanalysis rather than targeted gene testing, as lab reanalysis triggered no financial charge to the patient. In this case, the specific variant for confirmation would be communicated to the reference laboratory that completed the initial ES/GS. If the patient’s reanalysis had already been utilized, the clinician would initiate a prior authorization process for the specific variant confirmation following a standardized workflow. BCH’s International Office was engaged by clinicians in cases for whom the patient’s primary address was outside of the United States. Pilot funds were allocated to support variant confirmation in cases when prior authorization was denied or unavailable.

## Results

### Diagnostic trends and correlates in clinically ordered ES/GS at BCH

We reviewed the results from the diagnostic testing conducted by GeneDx (“testing laboratory”). From 2021 through 2024, an average of 996 probands per year had clinical sequencing genomic data returned to BCH (ranging from 866 to 1,157) (Figure S1C). In 2025, the return of genomic data began growing more rapidly, with 1,134 probands’ data returned by May 1, accompanied by an increase in GS vs. ES. Review of reported variants included ES/GS cases for which variants had been returned in a structured and interpretable format (*n* = 6,045) and did not include other cases such as those sequenced at Claritas (Table 1). A total of 21.6% (*n* = 1,303) of these probands received a causative P/LP variant from the analysis; 43.2% (*n* = 2,677) of probands had no reported variants and 34.2% (*n* = 2,065) had variants that were VUS or P/LP but not causative for the patient’s disease. The 78.4% (*n* = 4,742) of probands without a causative P/LP variant could potentially benefit from reanalysis over time, which may identify a diagnosis, upgrade the interpretation of a variant(s) from VUS to P/LP or resolve a candidate or possibly associated gene into the causative gene for the patient’s disease. Additionally, while some studies demonstrated that ∼5% of diagnosed patients would typically be expected to have dual diagnoses involving two or more disease loci,^65^ in this cohort only 2.8% (*n* = 37) of probands with causative variants had causative variants in multiple genes, which may reflect underdiagnosis or ascertainment differences in this cohort.

We tested the association between the type of result and the patient’s age, sex, family structure, revised combined race and ethnicity category calculated by integrating self-reported race and ethnicity from the EHR,^38^ distance to BCH, type of health insurance, and median income of the zip code of residence to see if any variables were associated with having a finding on the clinical testing report (Figure S3). Across a total of 136 comparisons, 7 showed statistically significant differences (two-sided Fisher’s exact test, Benjamini-Hochberg corrected *p* < 0.001) (Table S1). For example, VUS were less often identified in trios (19% had VUS) compared with duos and singletons (29% had VUS). VUS were also less often identified in patients with private insurance (18%) compared with government insurance (24%), a difference likely influenced by associations between types of health insurance and the other variables (e.g., revised combined race and ethnicity category, family structure).

### Institutional baseline: Clinician-initiated reanalysis prior to PGR

To contextualize the clinical need addressed by PGR, we examined the frequency, uptake, and diagnostic impact of clinician-initiated laboratory reanalysis at BCH prior to implementation of the centralized PGR workflow. We reviewed the frequency of and impact from clinician-initiated reanalyses conducted at the behest of BCH clinicians by the testing laboratory and research reanalyses conducted by BCH researchers. Prior to the work described in this article, given the burden of reanalysis on individual clinicians, clinician-initiated lab reanalysis was performed infrequently (in 13.6% of patients, *n* = 852) and usually upon patient request and/or after a subsequent clinical encounter (Figure 1, blue box). Despite broad consensus among clinicians in the strategic working group that genomic data should be reanalyzed over time, real-world uptake remains low, with only 15% (754/4,975) of patients at BCH with documented plans for reanalysis (including probands whose original analysis was completed prior to the agreement with BCH, but had data returned after) actually receiving it. Overall, for probands with original testing performed in the last 6 years (since April 2019), clinician-initiated lab reanalysis had been completed for 6.9% of probands (*n* = 440), initiated by 279 different clinicians. This occurred on average 2.37 years (0.75 SD) after the initial analysis. These findings highlight the limited reach and scalability of clinician-initiated reanalysis under current practice and underscore the need for a centralized, proactive approach.

White, non-Hispanic/non-Latino revised combined race and ethnicity category patients were more likely (9%) than patients with other revised combined race and ethnicity categories (5%) to have had a clinician-initiated lab reanalysis, while patients with race/ethnicity not reported (unknown or decline to report) were less likely (3%) to have had a clinician-initiated lab reanalysis (Table S1). A total of 17% (*n* = 76) of those with clinician-initiated lab reanalysis had their report changed over time, with the majority (*n* = 64) having variants added or variants upgraded. Ten probands had variants that were downgraded or removed and two patients had a variant upgraded/added and a variant downgraded/removed (Table 1). Additionally, 16% of patients (*n* = 953) were enrolled in a Mendelian disease research study by 103 clinician-scientists, where their data were reanalyzed by research teams (3.6% of patients, *n* = 205, had both clinical and research reanalysis performed). Although research reanalysis can help close the gap, a substantial fraction of clinically sequenced patients could benefit from a centralized proactive approach.

### Semi-automated identification of prioritized variants

We developed a scalable PGR bioinformatics pipeline and centralized review process (Figure 2). The pipeline utilizes the established CRDC infrastructure,^32^^,^^33^ which includes variant calling, variant annotation and filtering, and collecting HPO terms. The in-house tool, CFA, was developed on the GeneDx research platform^66^^,^^67^ and applies further annotation and stringent filtering criteria. Specifically, CFA incorporates filters based on variant allele frequency and count, automated ACMG scoring,^37^ ClinVar^48^ classification, predicted variant effects, and inheritance patterns. Initial exploratory analyses of the clinical sequencing data reviewed variants and genes that had been added to ClinVar or OMIM^44^ after the original analysis date and had some phenotype overlap, as well as variants in genes that scored highly in association with the patient’s phenotype according to AMELIE^52^ and HPO3.^49^^,^^50^

To formalize and automate variant prioritization, we developed and trained a VS-NN algorithm, with 43 characteristics (Figure 2). We evaluated the performance of the VS-NN algorithm using variants reported by the testing laboratory as a validation dataset. Variants with prediction scores of 0.5 or higher were flagged as a threshold of ≥0.5 yielded the optimal balance of precision and recall (Figure S4A). TP variants were defined as variants reported by the testing laboratory that received a score above 0.5 from the VS-NN algorithm. FN variants were defined as variants reported by the testing laboratory that received a score below 0.5 from the VS-NN algorithm. False positive variants were defined as variants not reported by the testing laboratory that received a score above 0.5 from the VS-NN algorithm. We observed an area under the receiver operating characteristic curve (AUC) of 0.99 for P/LP variants and 0.94 for VUS (Figure S4B). While AUC provides a general measure of discriminative ability, it can be disproportionately influenced by the majority class, particularly in imbalanced datasets like ours where negative cases significantly outnumber positive ones. To address this, we further assessed model specificity and sensitivity. The model demonstrated strong precision and recall for P/LP variants at 74% and 83%, respectively. However, precision and recall for VUS were notably lower at 57% and 32%, suggesting limited performance in detecting VUS and highlighting an area for potential model improvement.

To better understand the performance of the VS-NN algorithm, we further examined the distinguishing characteristics among the TP and FN variants (Figure S5; Table S2). Compared with FN variants, TP variants had a lower allele frequency in gnomAD and across CRDC participants; a higher S_coef_, S_het_, HPO3 score, and AMELIE score; more HPO matches with information content cutoff of two; more likely to be classified as P/LP by the automated ACMG scoring, exhibiting *de novo* inheritance in dominant genes, they are less likely to have conflicting classification in ClinVar. TP P/LP variants also had a higher pLI, were more likely to be in OMIM dominant genes and pathogenic in ClinVar, and less likely to be part of a compound heterozygous variation or biallelic in proband only cases than FN P/LP variants. Whereas TP VUS had higher CADD PHRED scores, REVEL scores, AlphaMissense scores, MisFit D, MisFit S, HPO match, and HPO match with information content cutoff of four, they were less likely to be absent in ClinVar and more likely to be ClinVar VUS. Overall, these results show that rare variants with stronger *in silico* predictions and better phenotype matches are more likely to receive higher VS-NN scores, consistent with expectations. At the same time, the findings highlight the complexity of automating variant classification, pointing to opportunities for further model refinement.

We also reviewed the impact of providing the VS-NN algorithm with different subsets of HPO terms using the TP/FN validation cases, as a proxy for simulating phenotype changes over time. We compared using only HPO terms manually annotated by clinicians and researchers with including HPO terms automatically extracted from the clinical notes with NLP (Table S3). The advantage of including NLP-extracted HPO terms is that patient phenotypes can change over time and that NLP extraction enables a scalable method to capture this change, which is why we trained the classifier on the combined HPO terms. There was no statistically significant change for including the NLP-extracted HPO terms on the VS-NN algorithm score, supporting the feasibility of integrating dynamic phenotype data at scale. Furthermore, we simulated the impact of changing phenotypes over time by analyzing how pruning HPO terms from the manually annotated HPO terms changed the VS-NN algorithm score. We observed a significant decrease (10%) in the score for TP and no change for FN variants with pruning the HPO terms. When evaluating the general performance of the scoring algorithm on all variants input for these patients, there was no difference between scores for any group of HPO terms (combined, manual only, pruned), indicating that the change in performance was specific for relevant, high-scoring variants rather than a global score shift.

An internal two-pass review was utilized by the centralized working group. Flagged variants were reviewed by a bioinformatician who assessed phenotypic match, mode of inheritance, variant quality, *in silico* predictions, and frequency in gnomAD v.4.^42^ Given the high specificity of the VS-NN algorithm implemented during the initial pilot, only 253 patients had results (310 variants). Manual review by the bioinformatician winnowed this down to 53 patients with 57 variants. Variants were rejected because there was a mismatch between the phenotypes reported for the gene and the patient (54%, *n* = 169), the mode of inheritance was not consistent with the disease inheritance pattern reported in OMIM (12%, *n* = 37), the reads in the BAM file did not pass manual quality assessment (8%, *n* = 25), or *in silico* predictions suggested a benign or likely benign classification (7%, *n* = 22). This review took on average 5 min per variant. A second pass review determined ACMG criteria and classification and performed a more in-depth review of phenotype match and molecular mechanism as needed, either adhering to published gene-disease validation and variant curation guidelines (e.g., ClinGen) when available or critical review of available literature on genotype-phenotype correlation to ensure variants communicated to clinicians would be most likely to be relevant to the proband’s phenotype. This second review averaged 3 min per variant. A total of 45 variants in 42 patients passed the second pass review; 16% of the variants that passed the first pass review (*n* = 9) were rejected as they were already documented in the EHR; these were all in the unknown classification/Claritas dataset and there was no structured data for variants returned for these cases. Five percent (*n* = 3) of the variants were rejected due to phenotype, with the second pass reviewer not considering the disease phenotype and patient phenotype closely enough matched to warrant reporting. An internal report was written by the centralized working group (Figure S6), which averaged 20 min per proband.

### Centralized PGR pilot results

**Figure 4:**
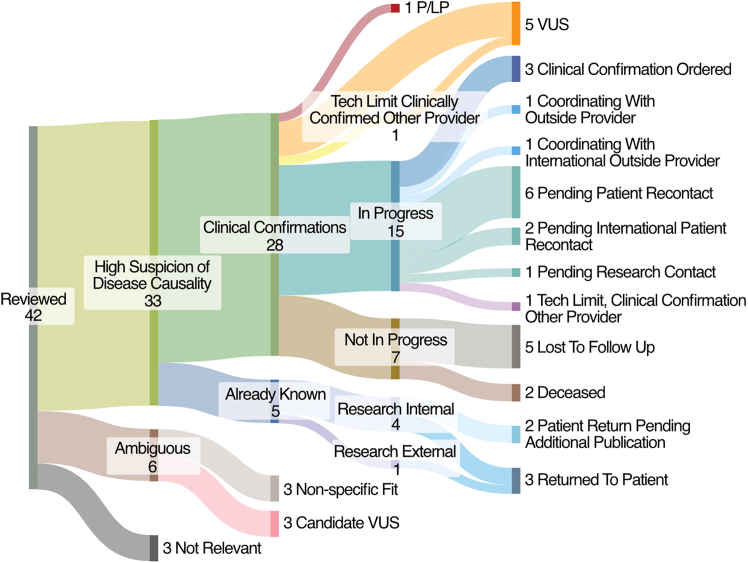
Early results This Sankey diagram visualizes the results from the initial batch of analyses that were shared with clinicians. Variants were reviewed in the context of the patient’s phenotype and clinicians determined whether each variant was a potential clinical diagnosis, not relevant to the phenotype or ambiguous. Variants were further subtyped to already known or requiring clinical confirmation. Results were annotated P/LP, VUS, in progress, or not in progress. In progress cases were subtyped to clinical confirmation ordered, coordinating with outside providers, pending patient recontact, and technical limitation from GeneDx/reflexed to alternative reference laboratory. Not in progress cases were subtyped to lost-to-follow-up and deceased. This figure was created using SankeyMATIC.^33^

For the initial pilot implementation of the PGR workflow, downstream variant prioritization and manual review focused on 1,639 patients who did not have reported variants identified by the testing laboratory and 505 patients where the variant reports were unknown using the PGR workflow (PGR bioinformatics pipeline followed by the clinical process plan, Figure 2), we then tracked the results of this round of analyses. Twenty-three unique providers were involved in communications about the variants that passed the internal two-pass review process, reflecting downstream clinical follow-up within the defined workflow (Figure 3). Clinicians were only asked to review the small number of prioritized variants which required modest effort and was similar to the skills required to assess a clinical report. Clinicians identified 33/42 probands who had variants identified by the centralized working group as having variants with a high suspicion of disease causality. Of the other nine probands, three had variants that were considered to be candidate VUS predicted not to positively impact patient care while the rest did not have a strong enough fit to the phenotype (Figure 4). Different providers had to be nominated due to provider turnover in 24 of the cases. Twenty-two patients had been sequenced at Claritas, which closed in 2017 and did not offer subsequent reanalysis. Even among GeneDx cases (*n* = 20), five had already utilized their clinical reanalysis. Of the GeneDx cases in which the variant was considered by the clinician to have a high suspicion of disease causality (*n* = 14), nine needed clinical reanalysis for confirmatory testing. Two had already used the clinical reanalysis, triggering the need for single-gene confirmation and prior authorization. Reasons that confirmatory testing was not pursued for some probands included variants already being known due to research (*n* = 5) and not having reports uploaded to the EHR (three were returned to patients via research studies, two were awaiting publication of natural history studies, which would enable stronger variant classification); patients lost to follow-up (*n* = 5); and patients who died (*n* = 2). Additional complexities included technical limitations with specific loci preventing confirmation and reporting of certain variants by one laboratory, but were able to be tested at another reference laboratory (*n* = 2). To date, one proband received a P/LP variant report, 5 received reports with VUS in candidate genes that were likely to positively impact patient care, and the 15 remaining probands where the clinician agreed with the diagnosis remain in progress. Under the conservative criteria used in this initial pilot, the PGR workflow identified candidate variants in 42 of 2,144 reviewed cases, with clinicians determining that 33 represented a high suspicion of disease causality.

To examine whether any variables were associated with having a finding through the PGR workflow, we tested if there was any difference in the patient’s age, sex, family structure, revised combined race and ethnicity category, distance to BCH, type of health insurance, or median income of the zip code of residence between patients who had variants that were input into the VS-NN algorithm, patients with variants that passed the VS-NN algorithm score threshold, patients with variants that passed either step of review, and patients with variants considered to be high suspicion of disease causality or candidate VUS. Given that such a large fraction of the patients with pilot PGR process results were sequenced at Claritas between 2015 and 2017 and the changing nature of clinical guidelines around sequencing, we stratified these analyses by laboratory. With this stratification, there were no statistically significant differences between the ratios of any of these variables.

Identified variants were analyzed according to Robertson et al., who categorized mechanisms driving the increases in diagnostic yield in reanalysis studies.^8^ Two of the VUS were not included in the original report due to limited clinical overlap at the time of the original test (“gathering of detailed phenotype information and/or evolution of the patient phenotype”). Three variant identifications were classified as due to technical limitations (“generation of additional genomic data and/or examination of additional variant types”): the two that were reflexed to another reference laboratory and one VUS diagnosis that was in a region that was not called by the clinical laboratory’s pipelines as it had been identified as a low coverage region. Thirteen had variant identifications due to the “discovery of gene-disease/variant-disease associations” that were reported in OMIM,^44^ GenCC,^64^ or ClinGen^68^ after the date of original analysis, including nine that had been sequenced at Claritas and four that had been sequenced at GeneDx.

## Discussion

In this article we describe the development and implementation of a PGR workflow for clinical sequencing data at a single children’s hospital that is centralized, semi-automated and clinically integrated and designed to bridge clinical and laboratory data silos and empower clinicians to engage in ongoing variant interpretation (Figure 1). Clinician-initiated reanalysis is included as an institutional comparator to contextualize the need for a centralized PGR workflow. Prior work shows that faster integration of comprehensive clinical data and up-to-date gene-disease associations can increase diagnostic yield by 15% while avoiding unnecessary testing.^69^ Building on this evidence, we introduce an institutional model for clinical genomic medicine that embeds reanalysis directly into routine care. Leveraging infrastructure established by the CRDC, we integrated raw genomic data and evolving phenotype information from over 6,279 patients and their families, enabling scalable and clinician integrated reanalysis. The almost 80% of clinically sequenced patients at BCH without known causative pathogenic variants stand to benefit from systematic reanalysis that could identify variants, and all may benefit from automatically updating the classification of previously reported variants and identifying second loci that contribute to disease. The PGR workflow democratized access to reanalysis regardless of ordering provider, as sequencing data from all clinicians at BCH who have sent ES/GS sequencing were included in the PGR bioinformatic pipelines. A centralized working group oversaw variant prioritization using a combination of automated machine learning tools and expert review, resulting in the identification of potential diagnoses, some with treatment implications. Working through the process of returning the initial 42 patients’ identified candidate variants to the clinicians and, where appropriate, the patients, revealed the challenges and impact of adopting centralized institutional genomic reanalysis. In this initial pilot, the observed reanalysis yield was 2% using conservative filtering and review criteria, reflecting an intentionally high specificity approach.

Implementation of the CRDC infrastructure at BCH enabled integration of clinically returned genomic data and metadata, exemplifying how the research framework can be scalably repurposed to support clinical care. The transfer of data from the testing laboratory to BCH required sustained coordination between the two organizations, including regular communication to ensure accurate transformation of genomic data into a BCH-aligned schema. As clinical process streamlining led to substantial increases in the frequency of ES and GS (84 probands sequenced per month from 2021 to 2024, 248 probands sequenced per month beginning in 2025), the data transfer processes were scalable. This harmonization enabled efficient querying of previously siloed reported variants^70^ for IRB-approved research and preparatory to research activities. Critically, patients with existing clinical sequencing no longer required research-generated ES/GS, yielding substantial cost savings to the CRDC^33^ and other research studies (the total amount spent on processing and storing clinical sequencing data in the CRDC infrastructure is only about a third of the cost savings from reflexing clinically ordered sequencing data to research use). The CFA platform, originally built for research, was repurposed to deploy a centralized variant prioritization system with minimal overhead, illustrating that institutions with embedded research capacity can provide clinical insights by repurposing tools already at hand, enabling real-time use of clinical genomic data to inform care.

Despite promising early results from the PGR bioinformatics pipeline, several limitations have emerged that inform ongoing refinement. The VS-NN algorithm demonstrated high specificity but limited sensitivity, identifying candidate variants in only 1.8% of patients (Figure 1; Table 1). This low yield can be attributed to conservative filtering thresholds, a deliberate prioritization of precision over recall, and dependence on strong algorithmic phenotype matching using tools such as AMELIE and HPO3. When stratifying diagnostic yield by the time since sequencing or data return to BCH, we observed an increase in the percentage of patients receiving a diagnosis from reanalysis as the interval lengthened (Table 1), consistent with prior reports. Notably, the current workflow excluded patients with previously reported variants and did not include any algorithmic mechanism to identify those eligible for variant reclassification, both of which represent additional opportunities for diagnostic yield and potential impact on patient care.

Comparison with other large-scale efforts provides additional context. The diagnostic yield of the PGR pipeline is comparable with that of the 100,000 Genomes Project^10^ (100kGP), but lower than that of Solve-RD,^24^ where more variants per individual (mean = 8) were returned to task forces for review. In contrast, we returned a mean of 1.2 variants per patient, whereas 100kGP returned approximately 1.6. When including VUS, the VS-NN algorithm achieved a recall of 56% and precision of 80%, compared with 84% and 57% in 100kGP’s Exomiser pipeline, respectively, highlighting our more conservative approach. However, when restricted to variants classified as P/LP by both return status and automated ACMG scoring, similar to the optimal settings used by 100kGP, our recall and precision were comparable (recall: 90% vs. 82%; precision: 81% vs. 88%; VS-NN vs. 100kGP, respectively). These benchmarks suggest that integration of lessons from pipelines such as Exomiser could improve sensitivity, particularly for VUS detection.

Manual review during the two-pass prioritization process illuminated clear opportunities for algorithmic tuning. Future improvements will include pruning features that fail to distinguish between TPs and FNs, enhancing features that discriminate between true and false positives, and refining allele frequency and inheritance filters. Adjustments to the phenotype-matching process, especially the weighting of evolving clinical phenotypes, may also increase sensitivity. Notably, a pruning analysis revealed that dynamic phenotypic updates significantly influenced classifier scores, but we did not assess how often those patient phenotypes changed over time. Future work will explore longitudinal HPO term evolution and its impact on reanalysis. Additionally, the current pipeline also restricts analysis to SNVs/InDels from the DRAGEN pipeline. Development is underway to expand support for other variant types, including structural variants and repeat expansions, which could further improve diagnostic rates.

The gap between the number of patients warranting genomic reanalysis and those receiving genomic reanalysis is large, reflecting a critical gap in current workflows, which rely on individual clinicians to initiate and navigate lab reanalysis requests, often limited by time, genomic expertise, and requiring patient prompting and partnership. As this reanalysis is currently clinically available only a single time per patient, there is uncertainty about optimal timing of this request. A centralized reanalysis model ameliorates some of these obstacles, improving access for all patients and all providers, not just those with advanced genetics training. It also enables the potential for repeated, longitudinal reanalysis, which is something current models rarely support. For example, commercial labs may offer only one reanalysis as part of the original ordered testing and require prior authorization for additional reanalysis. By embedding reanalysis into institutional infrastructure, genomic data become a living asset, capable of delivering diagnoses over time and reshaping care for previously undiagnosed patients.

The initial pilot implementation phase revealed critical real-world challenges in deploying centralized reanalysis. Many early cases originated from sequencing at Claritas (2015–2017) with patients who had not been seen clinically for several years at our institution and often no longer had an identifiable provider affiliated with the institution. This made recontact difficult. Unlike testing sent to other labs, these Claritas cases lacked access to free reanalysis and required sample collection for confirmation, adding logistical and financial hurdles. To address costs, limited funds were allocated to support single-gene confirmatory testing when reanalysis or insurance coverage was unavailable or denied. Additional complexities included classification discrepancies between the testing laboratory and the centralized working group, challenges confirming variants (necessitating alternative testing in two cases), and international patients or patients who had died, complicating recontact and result disclosure. Although the planned process was intended to involve the primary clinician who had followed the patient/ordered the initial ES/GS, in practice it was often the case that clinicians with genetics expertise had to become involved to facilitate the process. In the Department of Neurology, this led to substantial revision of the initially proposed workflow (see Figure S7). Overall, the PGR bioinformatics pipeline alleviates the need for clinicians to manually identify variants and provides baseline analysis for all cases; however, there still exists substantial work for clinicians and departments in vetting the clinical correlation of the variants, identifying a laboratory that can confirm the findings, obtaining insurance authorization for confirmation, communicating effectively with families throughout the confirmation process and managing the return of results. Ongoing optimization efforts include refining the communication protocols, and workflow logic through iterative case-by-case resolution to handle the variety of clinical scenarios encountered, laying the groundwork for a more resilient and scalable reanalysis process. Future efforts will evaluate diagnostic yield, clinical utility, and patient/provider impact, alongside an analysis of cost per diagnosis. Ensuring sustainability will require ongoing optimization, but the potential to close the diagnostic gap and streamline care makes this a powerful step forward in the evolution of genomic medicine. Our centralized process is designed to provide more equitable care which starts with an accurate diagnosis. As therapeutic options increase, the value of a diagnosis will increase, and it will be critical to make diagnoses as early as possible while therapies are most beneficial. We have recently entered the second phase of the project, initiating the iterative application of the PGR workflow across a broader cohort. Early analyses have already uncovered additional candidate variants, both in patients from the initial cohort as well as in individuals whose data were only included in the second phase of the project, as well as patients with variants that are eligible for reclassification. Importantly, we do not propose that the PGR workflow replace existing clinician-initiated or research-based reanalysis workflows. Rather, it is designed to complement these efforts by proactively identifying and returning findings that may otherwise be overlooked. However, we acknowledge that this model may be uniquely suited to institutions with specific capabilities. BCH benefits from a confluence of enabling conditions: a high volume of clinical sequencing sent to a single commercial laboratory, an established raw data repatriation pipeline, robust in-house bioinformatics and variant interpretation expertise, and a mature research informatics infrastructure. These factors enabled integration of the PGR workflow with minimal overhead and significant cost savings by reducing the need for redundant sequencing in research settings. For institutions without comparable resources, particularly those lacking a centralized research infrastructure or internal genomics expertise, the costs of developing and maintaining such a system may be prohibitive.

Moreover, our experience reflects a healthcare model prevalent in the U.S. and some other countries, where clinical genomic testing is often outsourced to commercial laboratories. This stands in contrast to healthcare systems with centralized or in-house genomic testing infrastructure, which operate under different constraints and incentives. We also benefited from sending nearly all testing to a single sequencing provider, simplifying integration. Scaling this model across multiple testing laboratories would require substantial additional development, although ongoing standardization efforts within EHR platforms such as Epic may facilitate broader implementation in the future. While the full PGR model may be best suited to high-volume, research-oriented institutions with existing genomic infrastructure, key components are broadly transferable. Structured variant triage processes, integration of longitudinal phenotype data, and centralized, clinician-facing tools can improve scalability and clinician engagement across varied care settings. A core strength of this model is that it reduces the burden on frontline providers by embedding reanalysis into institutional workflows without requiring additional clinician effort beyond standard care. Looking ahead, the PGR pipeline will be deployed regularly, biannually for the general patient population and more frequently (e.g., monthly) for high-priority groups such as NICU patients, offering a scalable approach to integrating dynamic genomic interpretation into routine clinical practice.

In summary, our experience demonstrates that a PGR workflow that is centralized, semi-automated, and clinically integrated is both feasible and impactful in a real-world healthcare setting. By integrating genomic and clinical data within a scalable institutional framework, we were able to systematically identify variants of interest, support reclassification of existing findings, and return diagnoses that may otherwise have been missed. This model addresses persistent obstacles to reanalysis, limited clinician capacity and time, and siloed data, by embedding reanalysis directly into the clinical workflow and supporting it with semi-automated tools and structured team-based processes. Looking ahead, other hospitals that care for large numbers of patients with rare and evolving phenotypes, and that embrace research-informed care, may also be positioned to implement similar centralized models. Reanalysis can be performed within single institutions or potentially can be supported centrally across institutions with federated data. Automated reanalysis has the potential to democratize access to genomic insight, shift genetic testing from a static to a dynamic and iterative process, and enable genomic medicine to scale in service of continuous diagnostic improvement and precision care.

## Data and code availability

• The VS-NN algorithm code generated during this study is available on GitHub: https://github.com/BCH-RC/PGR_VS_NN.
• Genomic data from research-consented patients analyzed during this study are available to BCH researchers and clinicians via the CRDC infrastructure and are also shared externally through the Genomic Information Commons project (https://www.genomicinformationcommons.org/).
• This study did not generate new publicly shareable datasets requiring deposition in a public repository. Given that the procedures described were conducted as part of a clinical quality improvement program, data from patients not consented to research are not publicly available.

## Acknowledgments

Funding provided by NIH/NHGRI R01HG010365, NIH/NHGRI U01HG011755, NIH/NINDS K08NS117891, and a Boston Children’s Hospital Office of Faculty Development/Basic & Clinical Translational Research Executive Committees Faculty Career Development Fellowship. The content is solely the responsibility of the authors and does not necessarily represent the official views of the NIH.

## Author contributions

Conception or design of the work, S.R., M.H.W., P.S., and W.K.C.; acquisition, S.R., A.G., B.S., L.S., A.M.D'G., M.I., J.C., J.S.S., A.K., L.R., A. Shimamura, O.B., S. Sacharow, J.S., S. Srivastava, A.R.K., A.A.E.H., A.L., H.O., J.J., E.R., B.F., A. Singh, C.L., R. Mallik, G.S., G.P., A.M., A.O’D.L., J.B., P.M.B., W.B., M.D., S.M., D.T.M., J.O., J. Petit, J. Picker, A.P., C.C., M.H.W., and W.K.C.; performance of analysis, S.R., W.S., C.F., T.K.T., J.H., R. McGonigle, and R. Mallik; figure generation, S.R., W.S., C.F., R. McGonigle, and C.C.; interpretation of data, S.R., W.S., C.F., and W.K.C.; creation of software, S.R., W.S., C.F., J.H., A. Singh, C.L., and R. Mallik; writing of the manuscript, S.R., C.F., P.S., and W.K.C.; analysis of reported variants, B.S., L.S., A.M.D'G., M.I., J.C., J.S.S., A.K., L.R., A. Shimamura, O.B., S. Sacharow, J.S., S. Srivastava, A.R.K., A.A.E.H., A.L., H.O., G.P., A.M., A.O’D.L., J.B., P.M.B., W.B., M.D., S.M., D.T.M., J.O., J. Petit, J. Picker, M.H.W., and W.K.C.; review and editing of the manuscript, S.R., W.S., C.F., T.K.T., J.H., R. McGonigle, A.G., B.S., L.S., A.M.D'G., M.I., J.C., J.S.S., A.K., L.R., A. Shimamura, O.B., S. Sacharow, J.S., S. Srivastava, A.R.K., A.A.E.H., A.L., H.O., J.J., E.R., B.F., A. Singh, C.L., R. Mallik, G.S., G.P., AM, A.O’D.L., J.B., P.M.B., W.B., M.D., S.M., D.T.M., J.O., J. Petit, J. Picker, A.P., C.C., M.H.W., P.S., and W.K.C. All authors read and approved the final manuscript.

## Declaration of interests

A.O’D.L. has consulted for Addition Therapeutics and received research support in the form of reagents from Pacific Biosciences. M.H.W. has consulted for Illumina and Sanofi and receives speaking honoraria from Illumina and GeneDx. W.K.C. received in kind support for research from Illumina and GeneDx. E.R., J.J., and B.F. are employees of and may own stock in GeneDx.

## Supplemental information

Document S1. Figures S1–S7 and Tables S1–S3

Document S2. Article plus supplemental information
