# Supplementary material for: Scaling genomic reanalysis to unlock diagnoses and transform rare disease care
Source: HGG Adv. 2026 Feb 18;7(2):100582. doi: 10.1016/j.xhgg.2026.100582 (PMC12993396; doi:10.1016/j.xhgg.2026.100582)
Supplement: Document S1. Figures S1–S7 and Tables S1–S3 [file mmc1.pdf]

HGGA, Volume 7

Supplemental information

Scaling genomic reanalysis to unlock diagnoses and transform rare disease care

Shira Rockowitz, Wanqing Shao, Courtney French, Tina K. Truong, Jacob Hagen, Rylee McGonigle, Alexa Geltzeiler, Beth Sheidley, Lacey Smith, Alissa M. D'Gama, Mira Irons, Janet Chou, Joan Stoler, Amy Kritzer, Lance Rodan, Akiko Shimamura, Olaf Bodamer, Stephanie Sacharow, Janet S. Soul, Siddharth Srivastava, Amy Roberts Kennedy, Aya Abu-El-Haija, Abbe Lai, Heather Olson, Jane Juusola, Erin Ryan, Bethany Friedman, Anupama Singh, Cliff Li, Rittika Mallik, Gwendolyn Strickland, Gillian Prinzing, Alisa Mo, Anne O'Donnell-Luria, Jeff Bolton, Philip M. Boone, William Brucker, Michael Duyzend, Sonal Mahida, David T. Miller, Jacklyn Omorodion, Jeanette Petit, Jonathan Picker, Annapurna Poduri, Colleen Carlston, Monica H. Wojcik, Piotr Sliz, and Wendy K. Chung

Supplemental Information

Table of Contents

- Supplemental Figures and Legends
  - Figure S1: Data processing and genomic ecosystem
  - Figure S2: Clinician genetic test ordering and preparedness to analyze genomic data
  - Figure S3: Demographics by analysis result
  - Figure S4: VS-NN algorithm performance
  - Figure S5: PGR VS-NN algorithm features at true positive and false negative variants in the validation dataset
  - Figure S6: Internal report
  - Figure S7: Neurology Current Workflow
- Supplemental Tables
  - Table S1: Statistical analysis of EHR metadata and reported variants
  - Table S2: Statistical analysis of PGR VS-NN algorithm features that are associated true positive and false negative variants in the validation dataset
  - Table S3: HPO Pruning Analysis

Supplemental Figures and Legends

Figure S1: Data processing and genomic ecosystem

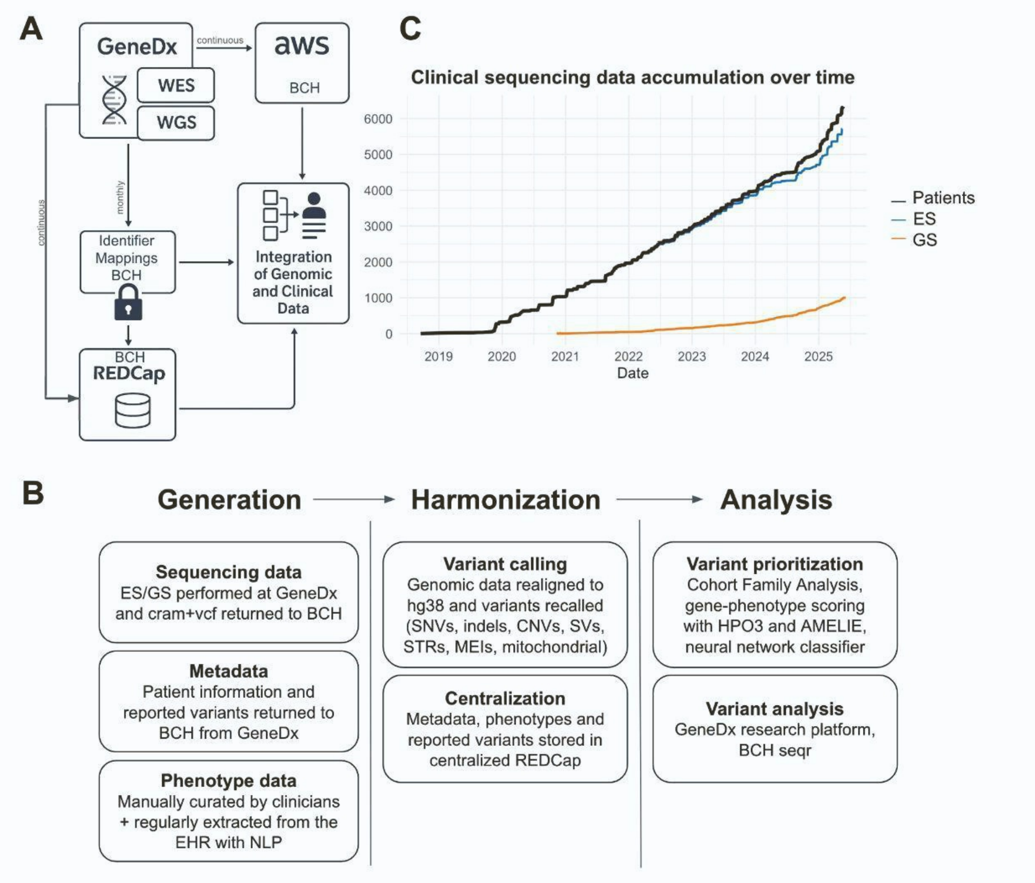

A) A robust data transfer and integration framework was established between GeneDx and BCH. Genomic data—including exome sequencing (ES) and genome sequencing (GS), excluding targeted exome sequencing—were delivered to BCH via Amazon Web Services (AWS) as sequencing was completed. In parallel, de-identified metadata for each case was systematically uploaded to REDCap. To support ongoing data linkage, GeneDx provided monthly encrypted mappings of accession numbers to identifiers. A custom extract-transform-load (ETL) process was developed, deployed, and iteratively refined to translate case-level metadata—where each additional family member or reanalysis review generated a new case—into an individual-centric format compatible with the BCH database architecture.

B) The existing CRDC data processing engine was adapted to support clinical reanalysis. The infrastructure enabled the recurring collection of HPO terms from the EHR via NLP, genomic data harmonization via secondary DRAGEN variant calling pipelines, and loading data into variant identification tools, allowing for scalable interpretation and centralized analytic tools that fostered clinician collaboration.

C) This plot tracks the increase in total number of patients sequenced at GeneDx whose data was returned to BCH over time. The breakdown between ES (blue) and GS (orange) is depicted.

Figure S2: Clinician genetic test ordering and preparedness to analyze genomic data

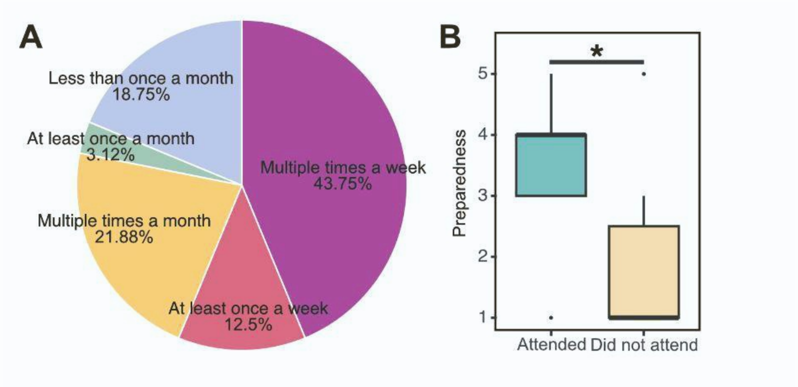

(A) Responses from 32 independent clinicians – attending physicians (n=13) physicians-in-training (n=7), genetic counselors (n=10), and nurse practitioners (n=2) – working in genetics and metabolism from the Division of Genetics and Genomics and the Department of Neurology on the frequency of ordering genetic testing: multiple times a week (n=14), at least once a week (n=4), multiple times a month (n=7), at least once a month (n=1), less than once a month (n = 6)
(B) Responses from these clinicians (six did not answer this question) on preparedness to analyze patient genomic data, stratified by the genome analysis tutorial attendance: attended (n=7), did not attend (n=19). Unpaired t-test p-value = 0.0069.

Figure S3: Demographics by analysis result

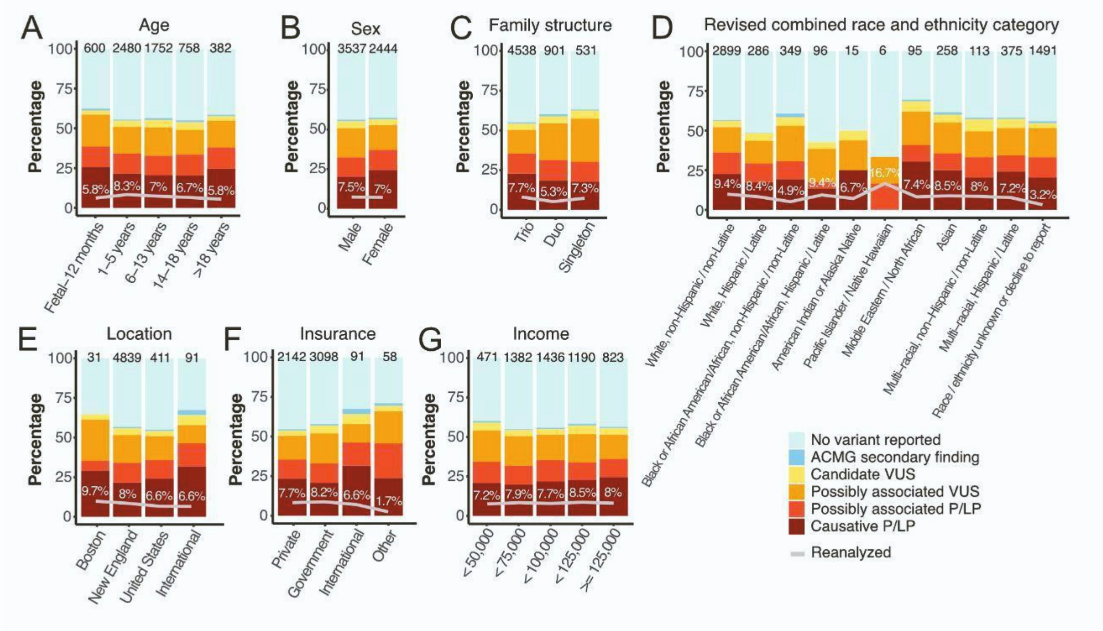

These barcharts show the breakdown of demographics: age (A), sex (B), family structure (C), revised combined race and ethnicity category as defined in Chopra *et. al.*[38] (D), location measured as distance from the hospital (E), type of health insurance (F), and median income of the zip code of residence (G). The percentage of probands who received GeneDx reanalysis is represented by the grey line.

Figure S4: VS-NN algorithm performance

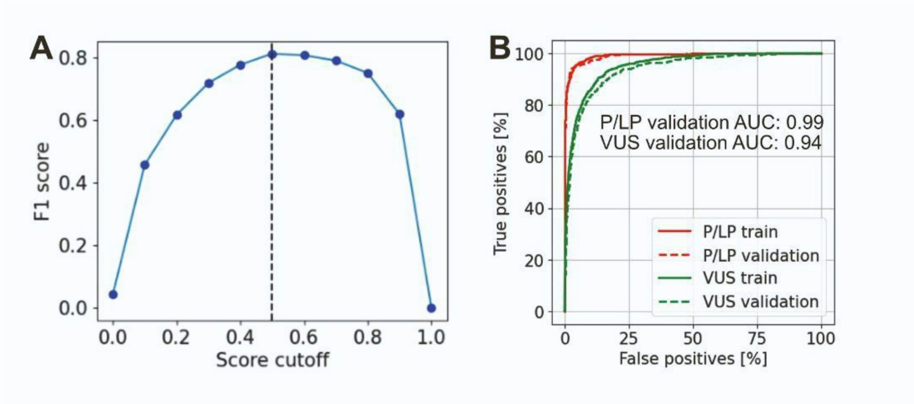

A) Distribution of F1 scores across prediction score thresholds in the validation dataset. A threshold of ≥ 0.5 yielded the optimal balance of precision and recall.
B) Receiver operating characteristic (ROC) curves and area under the curve (AUC) values for P/LP and VUS classification, evaluated on both training and validation datasets.

Figure S5: PGR VS-NN algorithm features at true positive and false negative variants in the validation dataset

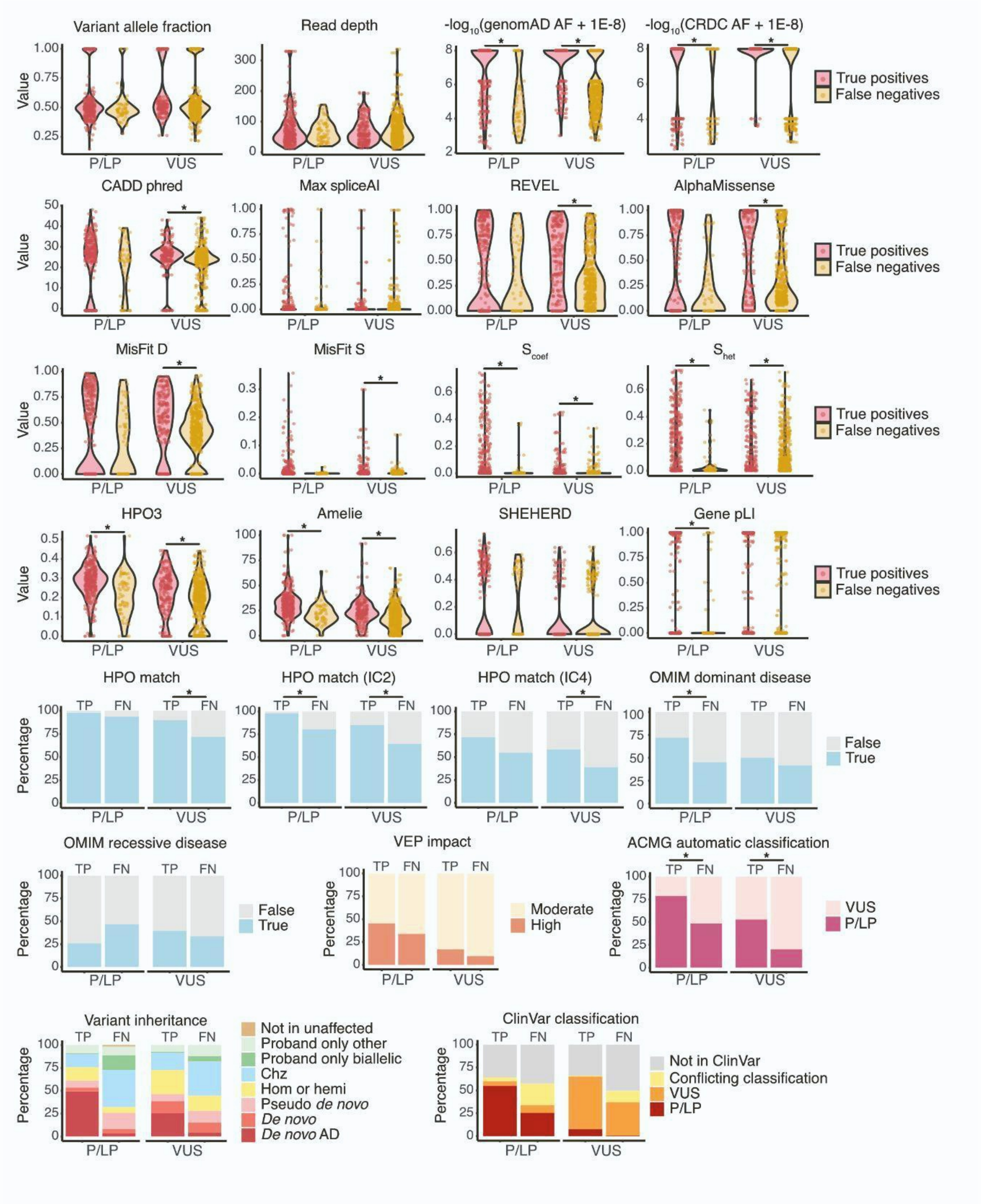

Violin plots and barcharts showing the distribution and breakdown of VS-NN algorithm features at VS-NN true positive (TP) and false negative (FN) variants in the validation dataset, stratified by clinical laboratory classification. Features include variant allele fraction, variant depth, gnomAD v4 frequency, CRDC internal frequency, CADD phred, SpliceAI, REVEL, AlphaMissense, MisFit D, MisFit S, S_{coef}, S_{het}, HPO3, Amelie, SHEPHERD, pLI, HPO match,

HPO match with information content 2 (IC2) cutoff, HPO match with information content 4 (IC4) cutoff, OMIM disease inheritance, VEP impact, ACMG automatic classification, variant inheritance and ClinVar classification. Asterisks were added for statistical tests with adjusted p-value < 0.001. Detailed test results are shown in Table S2.

Figure S6: Internal report

Not a clinical report, not to be returned to patient without clinical confirmation by reference laboratory (e.g., GeneDx)

Variants:

Gene	Variant	Condition	Zygosity	Inherited From	Classification
GENE	chr1:123456G>A NM_0012345: c.1234+2C>T	Autosomal Recessive Neurodevelopmental Disorder	Heterozygous	Mother	Likely Pathogenic
GENE	chr1:567890T>C, NM_0012345:c.5678C>G, NP_0012345:p.Asp123Asn	Autosomal Recessive Neurodevelopmental Disorder	Heterozygous	Father	Variant of Uncertain Significance

Summary: These variants are classified as **Likely Pathogenic** and **Variant of Uncertain Significance.**

Biallelic missense and truncating variants in GENE have been reported in ten cases in the literature (PMID: 12345, 67890, 45678) with neurodevelopmental disorder. This gene has been curated with strong evidence of disease association (GenCC HGNC:12345). These cases consistently exhibit a neurodevelopmental disorder characterized by global developmental delay, intellectual disability, and hypotonia. Additional common features include craniofacial anomalies, microcephaly, and seizures. Symptoms varied among reported cases. Functional studies in zebrafish models have demonstrated that loss of *gene* function leads to abnormal lens development and disrupted circadian rhythms, supporting the gene's role in neurological development.

ACMG Criteria:

chr1:123456G>A

- **PVS1:** null variant (nonsense, frameshift, canonical ±1 or 2 splice sites, initiation codon, single or multiexon deletion) in a gene where LOF is a known mechanism of disease
- **PM2**: This variant is at a low frequency in population databases (gnomAD v4.1)

chr1:567890T>C

- **PM2**: This variant is at a low frequency in population databases (gnomAD v4.1)
- **PP3**: Multiple lines of computational evidence support a deleterious effect on the gene or gene product (conservation, evolutionary, splicing impact, etc.)

Recommendations:

- Clinician phenotyping to assess whether the features of this condition match the patient's presentation.
- If you would like to return this result:
  - If sequencing was done at GeneDx, consider ordering a formal reanalysis and indicating this specific variant of interest
  - If sequencing was done outside of GeneDx, a single variant order will need to be placed with a new specimen (following usual clinical workflow with prior authorization)
    - If parents are available, consider confirming the variant in the parents as well so that inheritance can be established.
  - If you are experiencing issues with the clinical confirmation process, please respond to this email.

Example internal report generated following the template developed by the centralized working

group.

Figure S7: Neurology Current Workflow

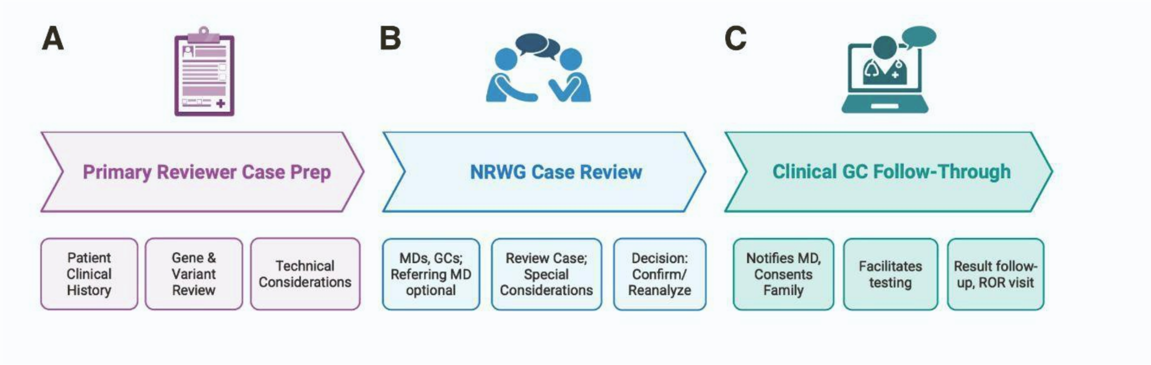

A) Preliminary review is conducted by a genetic counselor (GC). The GC collates data on the patient's phenotype, the gene and variant under consideration, and any technical considerations (such as whether a confirmation is possible at the designated lab or an alternative approach is required) for the Neurology Reanalysis Working Group (NRWG) to make a decision on confirmation. This includes medical, demographic and social histories in terms of recontact and potential for a return of result (ROR) visit.
B) NRWG reviews clinical & variant details, makes the decision as to whether there is sufficient evidence to support confirmation and return to family versus continued reanalysis. If a variant warrants confirmation, the NRWG assigns a Clinical GC to the case.
C) Clinical GC works with the family and clinical providers to pursue testing, collect any new samples that may be required, and follow-up on results. The clinical GC will participate in the ROR visit along with Neurogenetics MD to discuss results.

Supplemental Tables

Table S1: Statistical analysis of EHR metadata and reported variants

Comparison group 1	Comparison group 2	Group 1 percentage	Group 2 percentage	Adjusted P-value
VUS in trio families	VUS in other family structure groups	18.82%	29.47%	8.97E-15
VUS in duo families	VUS in other family structure groups	27.30%	20.32%	1.17E-04
VUS in singleton families	VUS in other family structure groups	33.15%	20.22%	2.81E-09
VUS in probands with private insurance	VUS in probands with other insurance	18.21%	23.81%	3.10E-05
VUS in probands with government insurance	VUS in probands with other insurance	23.98%	18.33%	2.67E-05

Reanalysis in White, non-Hispanic probands	Reanalysis in other revised combined race and ethnicity category groups	9.42%	5.35%	1.05E-07
Reanalysis in Unknown or decline to report probands	Reanalysis in other revised combined race and ethnicity category groups	3.22%	8.68%	6.11E-12

Results of two-sided Fisher's exact tests identifying demographic variables significantly associated with the outcome of interest. P-values were adjusted for multiple hypothesis testing using the Benjamini-Hochberg method. Only variables with an adjusted p-value < 0.001 are shown.

Table S2: Statistical analysis of PGR VS-NN algorithm features that are associated true positive and false negative variants in the validation dataset

Characteristics	Comparison group 1	Comparison group 2	Group 1 value	Group 2 value	Adjusted P-value
$-\log_{10}$(gnomAD AF + 1E-8)	P/LP TP	P/LP FN	6.83	5.16	2.25E-10
$-\log_{10}$(gnomAD AF + 1E-8)	VUS TP	VUS FN	7.16	5.80	4.12E-14
$-\log_{10}$(CRDC AF + 1E-8)	P/LP TP	P/LP FN	6.99	5.59	6.50E-07
$-\log_{10}$(CRDC AF + 1E-8)	VUS TP	VUS FN	7.57	6.84	2.85E-04
CADD phred	VUS TP	VUS FN	24.49	21.50	6.35E-06
REVEL	VUS TP	VUS FN	0.47	0.31	9.98E-05
AlphaMissense	VUS TP	VUS FN	0.57	0.29	5.03E-08
MisFit D	VUS TP	VUS FN	0.50	0.39	4.73E-05
MisFit S	VUS TP	VUS FN	0.01	0.00	1.32E-06
S_{coef}	P/LP TP	P/LP FN	0.13	0.02	3.75E-13
S_{coef}	VUS TP	VUS FN	0.05	0.01	6.22E-14
S_{het}	P/LP TP	P/LP FN	0.23	0.05	1.96E-09
S_{het}	VUS TP	VUS FN	0.20	0.12	2.23E-04
HPO3	P/LP TP	P/LP FN	0.28	0.22	3.03E-06
HPO3	VUS TP	VUS FN	0.23	0.16	4.23E-09
Amelie	P/LP TP	P/LP FN	31.79	21.43	1.96E-09
Amelie	VUS TP	VUS FN	24.82	18.09	1.18E-07
Gene pLI	P/LP TP	P/LP FN	0.61	0.16	1.55E-10
HPO match	VUS TP	VUS FN	89.55%	70.82%	1.29E-04
HPO match with IC2 cutoff	P/LP TP	P/LP FN	97.99%	80.65%	3.88E-05
HPO match with IC2 cutoff	VUS TP	VUS FN	85.07%	64.13%	8.16E-05
HPO match with IC4 cutoff	VUS TP	VUS FN	62.69%	38.60%	5.38E-05
OMIM dominant disease	P/LP TP	P/LP FN	72.13%	45.16%	5.17E-04

ACMG automatic classification P/LP	P/LP TP	P/LP FN	78.74%	48.39%	5.38E-05
ACMG automatic classification P/LP	VUS TP	VUS FN	52.24%	20.97%	6.10E-09
De novo inheritance in dominant genes	P/LP TP	P/LP FN	49.43%	3.23%	6.41E-12
De novo inheritance in dominant genes	VUS TP	VUS FN	23.88%	4.86%	3.44E-07
Compound heterozygous inheritance	P/LP TP	P/LP FN	14.66%	40.32%	1.74E-04
Biallelic inheritance in proband only cases	P/LP TP	P/LP FN	0.86%	16.13%	1.44E-05
Not in ClinVar	VUS TP	VUS FN	31.34%	53.23%	4.21E-04
Conflicting classification in ClinVar	P/LP TP	P/LP FN	4.31%	23.73%	2.01E-04
Conflicting classification in ClinVar	VUS TP	VUS FN	0.75%	11.38%	4.21E-04
VUS in ClinVar	VUS TP	VUS FN	60.45%	34.46%	3.25E-05
P/LP in ClinVar	P/LP TP	P/LP FN	54.89%	25.42%	4.49E-04

Results of two-sided Wilcoxon rank-sum tests and Fisher’s exact tests identifying PGR VS-NN characteristics significantly associated with the outcome of interest at VS-NN true positive (TP) and false negative (FN) variants in the validation dataset. P-values were adjusted for multiple hypothesis testing using the Benjamini-Hochberg method. Only variables with an adjusted p-value < 0.001 are shown.

Table S3: HPO Pruning Analysis

VS-NN algorithm score comparison group 1	VS-NN algorithm score comparison group 2	Adjusted P-value	Mean score difference
TP variants using NLP-extracted HPO terms combined with manual HPO terms	TP variants using only manual HPO terms	0.215	0.031
TP variants using only manual HPO terms	TP variants using pruned HPO terms	1.65E-07	0.095
FN variants using NLP-extracted HPO terms combined with manual HPO terms	FN variants using only manual HPO terms	0.629	0.007
FN variants using only manual HPO terms	FN variants using pruned HPO terms	0.004	0.043

Results of two-sided Wilcoxon rank-sum tests comparing VS-NN algorithm scores for true positive (TP) and false negative (FN) variants in the validation dataset using different sets of HPO terms. P-values were adjusted for multiple hypothesis testing using the Benjamini-Hochberg method.
